# Composition of the Cytochrome *c* Complex with Cardiolipin by Thermal Lens Spectrometry

**DOI:** 10.3390/molecules28062692

**Published:** 2023-03-16

**Authors:** Mikhail A. Proskurnin, Elena V. Proskurnina, Viktoriya R. Galimova, Andrei V. Alekseev, Ivan V. Mikheev, Yuri A. Vladimirov

**Affiliations:** 1 Analytical Chemistry Division, Chemistry Department, M.V. Lomonosov Moscow State University, d. 1, Str. 3, Lenin Hills, GSP-1 V-234, 119991 Moscow, Russia; blin3006@gmail.com; 2 Laboratory of Molecular Biology, Research Centre for Medical Genetics, 1 Moskvorechye St, 115522 Moscow, Russia; proskurnina@gmail.com; 3 Russian Research Institute of Aviation Materials, ul. Radio 17, 105005 Moscow, Russia; kvark-87@mail.ru; 4 Faculty of Basic Medicine, M.V. Lomonosov Moscow State University, Leninskie Gory, A, 119991 Moscow, Russia; yuvlad@mail.ru

**Keywords:** cytochrome *c*, cardiolipin, thermal lens spectrometry

## Abstract

Thermal lens spectrometry along with spectrophotometric titration were used to assess the composition of the complex of oxidized cytochrome *c* (ferricytochrome *c*) with 1,1′,2,2′-tetraoleyl cardiolipin, which plays a key role in the initiation of apoptosis. Spectrophotometric titration was carried out for micromolar concentrations at which the complex is mainly insoluble, to assess the residual concentration in the solution and to estimate the solubility of the complex. Thermal lens spectrometry was used as a method of molecular absorption spectroscopy, which has two advantages over conventional optical transmission spectroscopy: the higher sensitivity of absorbance measurements and the possibility of studying the light absorption by chromophores and heat transfer in complex systems, such as living cells or tissues. Thermal lens measurements were carried out at nanomolar concentrations, where the complex is mainly in solution, i.e., under the conditions of its direct measurements. From the thermal lens measurements, the ratios of cytochrome *c* and cardiolipin in the complex were 50 at pH 7.4; 30 at pH 6.8; and 10 at pH 5.5, which fit well to the spectrophotometric data. The molecular solubility of the complex at pH 6.8–7.4 was estimated as 30 µmol/L.

## 1. Introduction

The interaction between cardiolipin (CL) and the mitochondrial protein cytochrome *c* (Cyt *c*) is considered a key process in the early stages of the intrinsic pathway of apoptosis [1]. The selective interaction of cytochrome *c* with cardiolipin leads to the protein unfolding, the oxidation of cardiolipin, and the permeabilization of the mitochondrial membrane [2]. Proapoptotic properties of the Cyt *c*–CL complex suggest its anticancer effects. Indeed, Cyt *c*–CL nanoparticles sharply activated apoptosis in two A2780 human ovarian cancer cell lines, including those resistant to doxorubicin [3]. On the other hand, strategies for protection against apoptosis are developed using, e.g., flavonoids that affect the interaction of cytochrome *c* with cardiolipin [4]. Thus, the mechanism of interaction between cytochrome *c* and cardiolipin is being actively studied to control this critical process.

The proapoptotic effect of the Cyt *c*–CL complex is based on oxidase properties of cytochrome *c* [5,6]. The interaction of cytochrome *c* with natural and synthetic membranes is realized through electrostatic interactions, hydrogen bonds, and hydrophobic effects and includes conformational changes in both proteins and lipids [7,8]. As a key event, the Fe…Met80 bond is broken and a slit is formed, thanks to which heme becomes available for the reaction with H_2_O_2_ [9]. As a result, cytochrome *c* changes its redox properties and loses its ability of electron transfer, but acquires the properties of peroxidase [10], pseudolipoxygenase [5], and lipoperoxidase [10,11]. Due to the acquired oxidase properties, cytochrome *c* oxidizes cardiolipin, which leads to a change in the physicochemical properties of the membrane [12]. Moreover, cardiolipin hydroperoxide causes irreversible protein modifications and cross-linking of Cyt *c* [13].

The enzymatic characterization of peroxidase activity revealed the need to determine the critical threshold concentration of CL, which has a deep physiological significance for in vivo processes. The lipid-to-protein ratio (LPR) in Cyt *c*–CL is of fundamental importance for understanding the structure of the complex under various conditions. Previously, we found LPRs at which maximum peroxidase activity was achieved and proposed the schemes of reactions of the peroxidase cycle catalyzed by Cyt *c*–CL [9,10,14]. Within this framework, a primarily new idea has been put forward about the possible structure of Cyt *c*–CL based on the study of the properties of the water-insoluble precipitate of this complex formed by adding a small volume of a methanol solution of 1,1′2,2′-tetraoleyl cardiolipin (TOCL) to aqueous cytochrome *c* at high concentrations of the components [15]. Measurement of the ratio of the amount of cardiolipin added to cytochrome *c* to the decrease in the amount of cytochrome *c* remaining in the aqueous solution showed that the aqueous complex Cyt *c*–CL has a certain composition of (Cyt *c*):(TOCL)_n_, where *n* was found to be ca. 35 at a pH of 7.4. Analysis of the periodic peaks in small-angle X-ray scattering curves of the precipitate made it possible to conclude that it consists of regularly packed nanospheres with a diameter of 11.2 nm, in the center of which is a “molten globule” of Cyt *c* (size, 5.6 nm), and the outer side is formed by a monolayer of cardiolipin with charged heads bound to cytochrome *c*, while fatty-acid chains are directed outwards [15]. Later, Cyt *c*–CL in non-polar media (chloroform and hexane) were found [16,17,18].

Elmer-Dixon et al. studied the cardiolipin reaction with the A site (a locus that includes four residues of positively charged lysine located in close proximity to the D omega-loop, providing Met-80 for heme binding) for human and yeast cytochrome *c* [19]. Two structural rearrangements in cytochrome *c* were demonstrated. The first, mild rearrangement occurs on the outer side of the vesicle at an LPR of ca. 8–10:1. The second structural rearrangement is significantly more pronounced and occurs at a higher LPR in the range from 22 to 43. There, a significant change in the conformation of the protein occurs, because of which it acquires peroxidase properties. These studies were carried out at pH 8.0, since at pH~7, strong scattering of the formed Cyt *c*–CL interferes with the registration of absorption spectra [20,21]. Differences in the mechanisms of complex formation at low (0.5–10) and high (5–60) LPRs were confirmed elsewhere [22]. It was found that extended lipid anchorage and peripheral binding appear to prevail at low and high LPRs, respectively. Mohammadyani et al. showed using NMR, fluorescence spectroscopy, and in silico simulation that a fivefold excess of cardiolipin is required for complete cytochrome *c* binding to it. Simultaneous binding to two sites on two opposite sides of the heme leads to the opening of the heme slit for the substrate [23].

The disadvantage of spectrophotometric experiments of this system is that the composition of the precipitate is formed at relatively high concentrations of cytochrome *c* and cardiolipin. Therefore, we applied an essentially different method of titration of a cytochrome *c* solution with a lipid by thermal lens spectrometry (TLS) as this method is based on the photothermal effects of light absorption rather than transmission measurements [24,25]. TLS is based on non-radiative transitions of excited molecules caused by the absorbed part of the radiation passing through the sample. Thermal relaxation of the absorbed energy leads to heating of the sample and the formation of an uneven spatial profile of the refractive index (the thermal lens). By increasing the power of the excitation radiation, it is possible to achieve the same high sensitivity as in luminescent analysis, but for non-fluorescent molecules [24,25]. By thermal lensing, the cytochrome *c* detection limit is 100 nmol/L [26]. Thus, TLS allows detection of the formation of Cyt *c*–CL directly in the solution at a low concentration of cytochrome *c* and lipid (10 μM and below). In addition, thermal lens measurements are significantly less dependent on light scattering than conventional spectrophotometry and depend on the heat transfer properties [24,27], which is vital for such light-scattering samples as suspensions of Cyt *c*–CL hydrophobic nanospheres. This is especially important at neutral pHs, where strong scattering of the formed complexes of cytochrome *c* with cardiolipin interferes with the registration of absorption spectra [20].

The aim of this study was to assess the composition of the complex during the interaction of oxidized cytochrome *c* (ferricytochrome *c*) with 1,1′,2,2′-tetraoleyl cardiolipin at different pH values by thermal lens spectrometry and its comparison with spectrophotometry.

## 2. Results

### 2.1. Assessment of the Cyt c–TOCL Complex Composition by Spectrophotometric Titration

**Figure 1:**
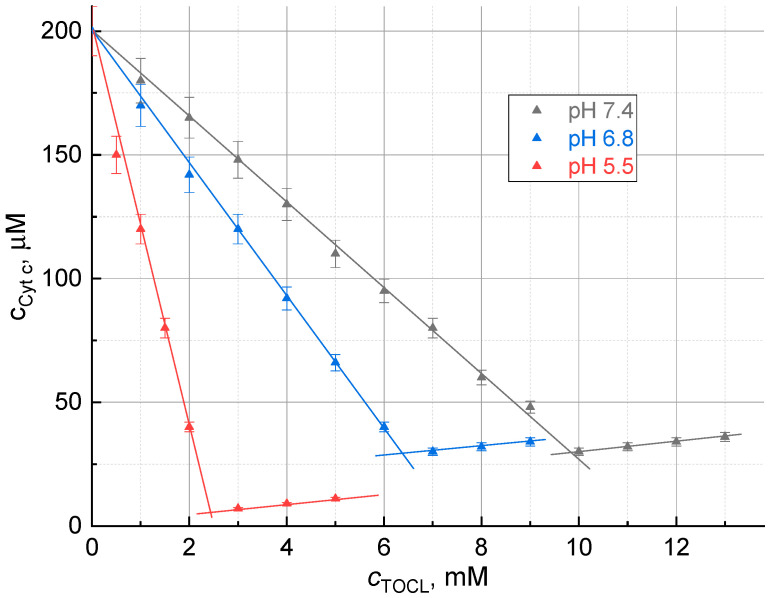
Dependence of the concentration of ferricytochrome *c* in the supernatant on the concentration of added cardiolipin at different pHs, 409 nm. Titration curves for pH 4.1 and 3.7 are not shown, they coincide with the curve at pH 5.5. At the inflection points, the complex in dissolved form is in equilibrium with the precipitate. After the inflection points, absorbance increases due to light scattering of excess cardiolipin appearing in solution.

A plot of the dependence of the concentration of ferricytochrome *c* in the supernatant on the amount of added TOCL (titration curve) at different pHs was built (Figure 1). Based on the existing data, a neutral pH (7.4), a pH of the intermembrane mitochondrial space (6.8), and a more acidic pH, corresponding to the pH on the surface of the inner mitochondrial membrane (3.9), are of greatest interest [28]. Thus, the pH range 3.7–7.4 was selected as characteristic to the acting conditions of cytochrome *c* in biological systems. Over this pH range, there is no coordination change in the heme iron and, thus, in ferricytochrome *c* redox properties [29].

The absorbance was measured at the Soret band (409 nm) [30], which is not affected by the complex formation [9]. The formation of the complex was checked by the absence of the weak absorption band at 695 nm, showing the ferricytochrome *c* structure change upon the interaction [9].

The ferricytochrome *c* concentration decreases to a certain point, and this decrease is directly proportional to the amount of cardiolipin added (Figure 1); thus, there is a certain stoichiometric ratio between ferricytochrome *c* and cardiolipin in the precipitate, TOCL:Cyt *c*. After this inflection point, the formation of the precipitate stops, and absorbance begins to increase due to light scattering of excess cardiolipin appearing in the solution (Figure 1).

The composition of the complex was determined by the point of intersection of the first phase of the titration curve with the abscissa axis. The quotient of dividing the concentration of TOCL at this point by the initial concentration of ferricytochrome *c* gives the TOCL:Cyt *c* ratio in the complex.

To determine the effect of pH on the composition of the complex, titration was carried out in an environment with a pH of 3.7 (a 20 mM phthalate buffer solution), 4.5, 5.5, 6.5, and 7.4 (a 20 mM phosphate buffer solution). The titration curves had a similar appearance, but the slope of the first part of the curve decreases with increasing pH. The composition of the complex differed (Figure 1). The composition of the complex and the values of solubility at different pHs are presented in Table 1. For comparison with thermal lens measurements, the concentrations of residual ferricytochrome *c* were measured at 409 and 515 nm (the working wavelength of the thermal lens excitation). In all the cases, the data correlated with the coefficients 0.999 or higher.

At the inflection points, the complex in dissolved form appears to be present, as evidenced by the absence of the absorption band at 695 nm [9]. Thus, the molecular solubility of the complex can be estimated from the absorbance at the inflection point (Table 1).

### 2.2. Assessment of the Complex Composition by Thermal Lens Spectrometry

The dependence of the thermal lens signal on the concentration of ferricytochrome *c* in the used buffer solution was linear for the concentration range 0.5–80 µM, the signal range, Equation (5), is 0.02–1.9. The detection limit by thermal lensing is 0.5 µM.

**Figure 2:**
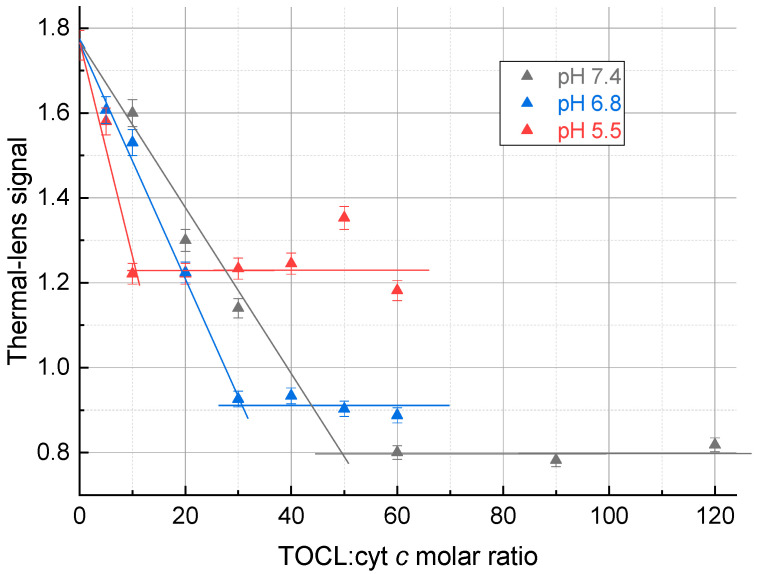
Thermal lens signal of aqueous solutions of the ferricytochrome *c* (4.0 µM) complex with TOCL on the TOCL:Cyt *c* molar ratio at different pHs. Excitation, 514.5 nm, 40 mW.

Thermal lens measurements were made at the same pHs but at 200-fold lower concentrations of the complex, which is lower than the solubility found by spectrophotometry (Table 1). The thermal lens signal decreased with an increase in the TOCL:Cyt *c* ratio down to a certain point (Figure 2). The plateau after the inflection point does not show low signals as the Cyt *c*–CL is in the solution, but the plateau signals differ significantly.

There is no increase in the thermal lens signal after the inflection point, as the scattering from excess TOCL does not affect the thermal lens signal. The thermal lens titration curves reached the equilibrium state with a TOCL:Cyt *c* ratio of 50 for a pH 7.4; 30 for a pH 6.8, and 10 for a pH 5.5 (Table 2).

## 3. Discussion

The idea of the application of thermal lens spectrometry for the assessment of the complex composition is that this method refers to both optical and thermal spectroscopy [27], i.e., the signal depends not only on light absorption but also on the thermal conductivity, density, thermal diffusivity, and thermal effusivity of the medium.

**Figure 3:**
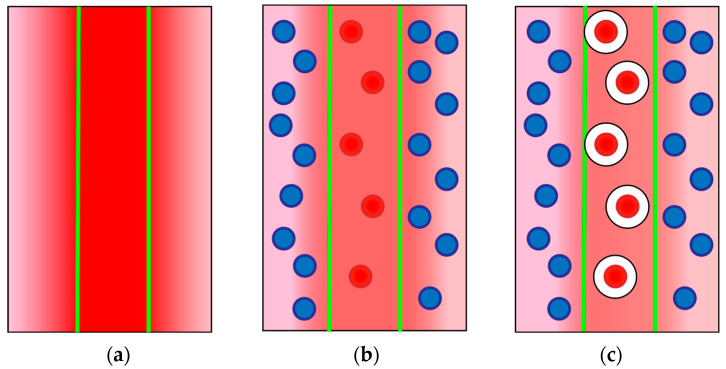
Formation of a temperature field in thermal lens experiments. The excitation beam, boundaries are denoted by green lines, irradiates the sample. In the case (**a**), a homogeneous solution of a chromophore, the heat transfers outside the irradiated zone in the centre and a temperature profile is formed. In the case (**b**), a solution of light-absorbing chromophore globules in a non-absorbing solution, the globules within the beam are heated and then the heat redistributes to the bulk solution. The overall temperature profile due to this redistribution is less pronounced than in the case (**a**). In the case (**c**), a solution of chromophore globules covered with some lowly thermally conductive substances in a non-absorbing solution, the heat transfer to the bulk of solution is hindered due to low thermal conductivity of the cover. The temperature field is less pronounced compared to the case (**b**). The formation of Cyt *c*–TOCL complex corresponds to a mix of cases (**b**,**c**).

For thermal lens measurements of dispersed media, the spatial distribution of the thermal properties of the medium is important. It has previously been shown that for dispersed systems, such as aqueous dispersions of fullerenes and nanodiamonds [31,32], as well as complex compounds and proteins (hemoglobin) [33], there is a decrease in the thermal lens signal compared to true solutions with the same solvent and the same light absorption. This is because the absorption of laser radiation leads to rapid local overheating of dispersed particles, but the temperature distribution profile caused by thermooptical phenomena throughout the sample has a smaller amplitude due to slower heat transfer from the heated dispersed particles to the dispersion medium (Figure 3a,b). If we compare two dispersed systems, with an increase in particle size (e.g., the formation of clusters of nanodiamonds [32] or with the formation of an albumin complex with an iron(II) complex with 1,10-phenanthroline [33]), the signal decreases.

Thus, based on these phenomena, for the Cyt *c*–TOCL complex, thermal lensing could be used for low concentrations of the components when the formation of the complex does not result in precipitation, while the absorbance of the solution does not change as the formation of the complex with TOCL does not result in a change at the Soret band [9]. However, the thermal lens signal should decrease due to the formation of larger particles in the solution.

This hypothesis was proven to be true (Figure 2) as the addition of TOCL decreases the signal, while after the equilibrium is attained, no change in the signal occurs. The plateau signals of the thermal lens titration curves also agree with this model; at low pHs, the number of TOCL molecules is lower, an increase in the particle size is smaller, and the total decrease is lower.

**Figure 4:**
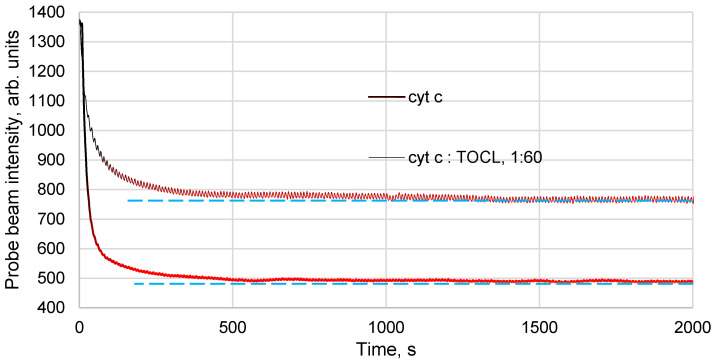
Thermal lens time-resolved signal (the blooming of the temperature field measured by a change in the intensity in the centre of the probe beam) of an aqueous solution of ferricytochrome *c* (4.0 µM) and the complex of ferricytochrome *c* of the same concentration with TOCL (Cyt *c*—TOCL; pH 7.4). Thermal lensing; 514.5 nm; 60 mW. Addition of TOCL results in a lower signal and a different development of the temperature field.

However, it should be noted that the situation with the formation of the Cyt *c*–TOCL complex is different from the cases of the formation of the albumin–ferroin complex or clusters in nanodiamond or fullerene solutions [33]. While in those cases we have chromophore particles of different sizes, here we have either a standalone cytochrome *c* molecule, which absorbs the photon (Figure 3b) or a Cyt *c* molecule surrounded by a shell of heat-insulating entities, hydrophobic fatty-acid chains of cardiolipin (Figure 3c). Thus, we have a different rate of heat transfer to the environment for the protein and its complex. The formation of Cyt *c*–TOCL nanoparticles leads to a significant change in the thermal properties of the dispersed system and a decrease in the thermal lens signal. The addition of TOCL results in a lower signal and a bit slower development of the temperature field compared to a ferricytochrome *c* solution with the same light absorption (Figure 4).

From these experiments, the conditions for thermal lens measurements providing the maximum accuracy of measurements, the stable steady-state thermal lens conditions (heating time, 1 s per cycle) were selected. The final precision of measurements by thermal lens spectrometry, despite significantly lower concentrations, is no less than by spectrophotometry (compare Table 1 and Table 2).

Thus, from both thermal lens and spectrophotometric measurements, the scheme of the complex formation (omitting protons) can be written as follows:

Cyt *c* + nCL ↔ Cyt *c*–CL_n_ (aq) ↔ Cyt *c*–CL_n_

For different pHs, in a neutral medium (pH 7.4), a ferricytochrome *c* molecule coordinates ca. 50–60 cardiolipin molecules. These data are generally consistent with previous results [19,22]. At a more acidic pH, a smaller excess of cardiolipin is required for the formation of the complex, but this excess is sufficient for a necessary change in the conformation of cytochrome *c* [23]. TLS data (Figure 2) are in good agreement with the results of spectrophotometric experiments (Figure 1). The value found is consistent with the assumed spherical structure of Cyt *c*-CL. From the Protein Database, the globule of this protein is an ellipsoid with a ratio of axes of 4.0 × 3.5 × 3.0 nm: its surface area is approximately equal to that of a 2 nm radius sphere, ca. 50 nm^2^. The surface of a partially molten globule of ferricytochrome *c* in the complex sphere of diameter 5.6 nm accounts for ca. 60 nm^2^. The data on the size of the phospholipid head of cardiolipin vary, but the cross-section of the three fatty-acid chains (if we assume the fourth buried in ferricytochrome *c*) is about 0.6 nm^2^ (estimated by ChemDraw^®^ software). Thus, the surface of protein in the case of the closest packing may hold up to 100 cardiolipin molecules. In fact, in the presence of double bonds in fatty-acid chains, the surface occupied by one molecule of cardiolipin is about twice as large as compared with that of close packing of the saturated hydrocarbon chains. Moreover, this surface depends on the conditions, especially on the medium pH. At a lower pH of 5.5, the TOCL:cyt *c* ratio decreases, and at pHs below 5.5 it remains approximately constant and low (10–12 molecules per a protein molecule, Figure 1 and Figure 2), although the molecular solubility of the complex decreases.

**Figure 5:**
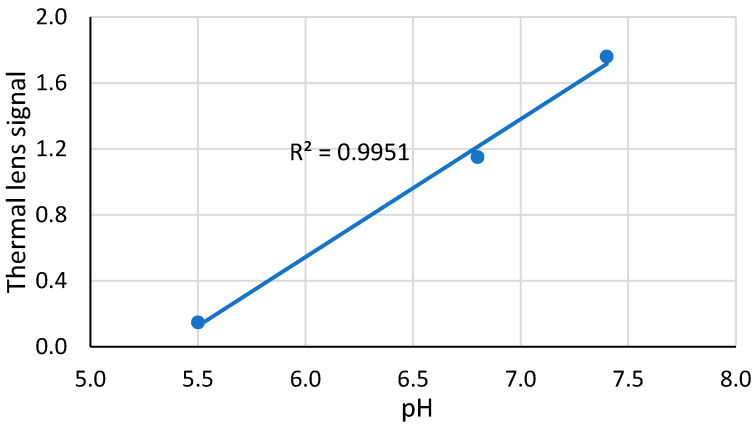
The dependence of the thermal lens signal of ferricytochrome *c*, 4.0 µM, after 4 h of preparation on pH; excitation, 514.5 nm, 40 mW.

The complex is precipitated at concentrations above 30 µmol·L^−1^ (at pH 7.4). At pH 5.5, molecular solubility drops down significantly (Table 1) due to a change in the unfolding properties of ferricytochrome *c* and a decrease in its stability with acidity [34]. In thermal lens experiments with freshly prepared ferricytochrome *c* solutions, this effect seems to be absent, although a significant drop in the thermal lens signal for low pHs is found after several hours (Figure 5); the signal for pH 7.4 changes insignificantly, although the complex compositions found for lower signals, differed insignificantly.

Such an almost linear dependence at concentrations lower than the molecular solubility also evidences a starting change in the unfolding and stability of ferricytochrome *c* [34] at pHs below 7.4, which confirms the results for molecular solubility by spectrophotometry. It seems important that a decrease in the signal at a rather high pH of 6.8 was not previously revealed by other techniques. Such behavior is important for understanding the thermodynamic properties of cytochrome *c* and could be a subject for separate study.

An interesting fact was a significantly decreased TOCL: Cyt *c* ratio at pH 5.5. With a further decrease in pH, no change in the stoichiometric ratio occurs. The interaction of cytochrome *c* with membranes is a complex phenomenon including conformational changes in both proteins and lipids [7,8]. Cytochrome *c* reacts with lipid membranes through electrostatic interactions, hydrogen bonds, and hydrophobic effects using four binding sites (A, C, N, and L). The variety of modes of interaction corresponds to a wide variety of specific reaction pathways [1]. The mechanism of interaction and the effect on heme are determined by lipid composition and pH [35]. At neutral pHs, hydrophobic interactions prevail, while at acidic pHs, cytochrome *c* binding is regulated by the interaction between head phosphate groups of cardiolipin and the amino acid side chains of the L-site, and NaCl has a strong influence [36,37]. The interaction also depends on the LPR. High LPRs promote electrostatic interactions [38]. Thus, to explain our results, we need to consider the reasons for a change in the charge at low pHs either on cytochrome *c* or cardiolipin.

At low pHs, cytochrome *c* exists in the native state (N), the denatured state (D), and in the state of molten globule (IIb and IIc states) [39]. The low-pH conformational equilibria of ferric yeast iso-1 cytochrome was studied by Battistuzzi et al. over a pH range from 2 to 7 [40]. Although there are certain differences between different cytochrome *c* isoforms, the key phase transitions appear to occur at more acidic pHs than 5.5.

Cardiolipin has a dimeric structure consisting of two phosphatidyl residues connected by a glycerol bridge and four acyl chains; therefore, it can carry two negative charges. It was previously reported that the p*K*_a_ values of these phosphate groups differ greatly: p*K*_a1_ = 2.8 and p*K*_a2_ = 7.5–9.5 [41]. However, Oloffson et al. performed pH-titration on 1,1′,2,2′-tetradecanoyl cardiolipin (C14:0) and 1,1′,2,2′-tetraoctadecanoyl cardiolipin (C18:1). Both compounds appeared to be strong dibasic acids with p*K*_a1_ = 2.15 and p*K*_a2_ about an order larger [42]. This means that at pH 5.5, phosphates are almost completely ionized. However, there is evidence that the concentration of protons at the lipid interface (on the membrane) differs from the bulk proton concentration. The pH at the interface of vesicles containing 25% cardiolipin, which corresponds in composition to the inner mitochondrial membrane, was estimated to be ca. 3.9, while the bulk pH was the same as the mitochondrial intermembrane space pH, 6.8 [43]. It is possible that at the bulk pH of 5.5, the surface pH may be about 2.5 or even lower, which causes the found changes in stoichiometry. The significantly more acidic surface pH may explain the decrease in the number of cardiolipin molecules bound to cytochrome *c*.

Our model implied the interaction in solutions; thus, the surface of the protein globule was completely available for the reaction. In reality, the interaction occurs with a membrane, so most studies used the liposome model [44]. The high LPR required an explanation. Brown et al. suggested that cytochrome *c* attaches to the membrane and gathers cardiolipin clusters around itself. As a result of subsequent studies, it was shown that cytochrome *c* undergoes significant conformational changes upon the interaction with cardiolipin [45,46] up to large-scale unfolding of the protein [47].

The interaction of cytochrome *c* with model membranes of cardiolipin/phosphatidylcholine was studied by Gorbenko et al. [48]. The authors concluded that the interaction mechanism is critically affected by pH. Estimations of the distance of the heme from the center of the bilayer indicate a shallow location of Cyt *c* in the bilayer at physiological pHs, while at pHs below 6.0, the protein tends to be incorporated into the membrane core. Domanov et al. showed that at a high concentration of cardiolipin in the membrane, the interaction cannot be described by the planar adsorption model and can be explained from the standpoint of the formation of non-bilayer structures and clusters, or the complete immersion of cytochrome *c* in the lipid bilayer [49]. Studies with giant liposomes and confocal fluorescence microscopy have shown that in the presence of cardiolipin and other anionic lipids, cytochrome *c* does not just attach to the surface but is able to wrap this layer around itself [50]. Thus, cytochrome *c* with cardiolipin forms spherical nanostructures [15,51]. Previously, we obtained complexes of cytochrome *c* with tetraoleyl cardiolipin in chloroform and *n*-hexane and determined the LPR as 77 ± 11. These results confirm the structure of the complex of cytochrome *c* with cardiolipin as a nanosphere [16]. Summing up, the nanosphere hypothesis allows us to compare data obtained on different models, both in solution and in liposomes or membranes and explain the good agreement between our results and the ratios obtained on liposomes and membranes.

In conclusion, we outline the prospects for further study. The inner mitochondrial membrane has a unique composition of proteins and phospholipids, the interaction of which is essential for mitochondrial function [52]. The lipid component contains major classes of phospholipids, including phosphatidylcholine, phosphatidylethanolamine, phosphatidylinositol, phosphatidylserine, phosphatidic acid, phosphatidylglycerol, and cardiolipin [53]. As well as cardiolipin, most of these lipids are capable of peroxidation, such as phosphatidylcholine [54,55], phosphatidylethanolamine [56,57], phosphatidylinositol [58], and phosphatidylserine [59]. Phosphatidic acid, conversely, showed an inhibitory effect on lipid peroxidation [60]. Although the interaction of cardiolipin with cytochrome *c* is considered a key event in mitochondrial apoptosis, positively charged cytochrome *c* reacts with other anionic lipids, primarily phosphatidylserine [61,62], but not with phosphatidylcholine and phosphatidylethanolamine [59]. The study of the cytochrome *c*–phosphatidylserine complex is of great interest for understanding the mechanisms of apoptosis [63]. Thermal lens spectrometry may be useful in solving this problem.

## 4. Materials and Methods

### 4.1. Reagents and Instrumentation

Ferricytochrome *c* from equine heart, 99%, *M* = 12,383 Da, for biochemistry (Sigma-Aldrich, St. Louis, MO, USA) was used. 1,1′,2,2′-tetraoleyl cardiolipin (TOCL) was from Avanti Polar Lipids Inc^®^ (Alabaster, AL, USA). Potassium dihydrogen phosphate, 99.995% for phosphate buffer solution preparation were from Fluka (Buchs, Switzerland). TOCL was dissolved in methanol (high-purity grade).

Water from a Milli Q water purification system (Millipore, Burlington, MA, USA) was used: specific resistance 18.2 MΩ·cm; Fe, 2 ppt; total ion amount, <0.2 ppb; TOC, <10 ppb; the own thermal lens signal, 0.004 ± 0.001. The glassware was washed with acetone followed by conc. nitric acid. The following reagents: 69% nitric and 37% hydrochloric acids (PA-ACS-ISO grade, Panreac, Barcelona, Spain), methanol (Sigma-Aldrich, St. Louis, MO, USA) high-purity grade, and phosphoric acid 85%, Riedel–de Haën (Seelze, Germany) were used throughout. All other reagents and solvents were of cp or higher grade and were used without further purification.

Spectrophotometric measurements were made using Agilent Cary 60 (Mulgrave, Australia) and SPECORD 200 (Analytic Jena, Hamburg, Germany) spectrophotometers with *l* = 10 mm, 0.3 cm^3^. The pH was measured by a pH 211-m (Hanna Instruments, Woonsocket, RI, USA) with a glass pH-selective electrode (precision, ±5%). Solutions were mixed with a Biosan MMS 3000 automixer (Biosan, Riga, Latvia) and a micro-stirrer.

### 4.2. Procedures

#### 4.2.1. Spectrophotometry

To determine the stoichiometric ratio of TOCL:Cyt *c*, spectrophotometric titration was used. To a 200 μM solution of ferricytochrome *c* a volume of 1.00 mL, 10 μL of a 100 mM TOCL solution was added (at a pH of 6.8 and 7.4) or 5 μL TOCL (at a pH of 3.7, 4.1, 5.5), while a red-brown precipitate of the Cyt *c*–TOCL complex was formed. After adding each portion of cardiolipin, the solution was centrifuged for 10 min at 10,000 rpm, the supernatant was separated from the sediment. The aliquot of the supernatant with a volume of 10 μL was brought with a buffer solution to a final volume of 1.000 mL, and the absorbance was measured in the Soret band, ε_409_ = 106,000 L/(mol·cm) [30]. The procedure for adding TOCL was repeated until the absorbance of the supernatant at 409 nm stopped dropping.

#### 4.2.2. Thermal Lensing

Titration of ferricytochrome *c* with cardiolipin was carried out as follows. To a 1.0 μM solution of ferricytochrome *c* a volume of 1.00 mL, different amounts of a 0.5 mM TOCL solution were added resulting in Cyt *c*:TOCL molar ratios of 1:10; 1:20; 1:30; 1:40; 1:50; 1:60; 1:90; 1:120; and 1:150. For each point, the thermal lens signal (Section 4.3 below) was measured at a wavelength of the excitation laser of 514.5 nm.

### 4.3. Thermal Lens Measurements

For thermal lens experiments, a coaxial two-laser spectrometer was used [33,64]. The thermal lens is induced in the cell by the radiation from an Innova 90-6 argon ion laser (Coherent, Palo Alto, CA, USA) with generation wavelength of 514.5 nm (TEM_00_ mode; maximum output radiation power, 1.5 W). As a probe laser, an HRP020 He–Ne laser (ThorLabs, Newton, NJ, USA) with 632.8 nm (TEM_00_-mode; radiation power, 2.0 mW) was used. Measurements were carried out at an optical path length of 0.2 cm. Other parameters are summed up in Table 3.

A measurement of the transient (time-resolved) thermal lens signal *ϑ*(*t*) for an excitation on/off cycle of a modulated continuous-wave (cw) excitation beam was calculated as a relative change in the probe beam intensity at a far-field detector plane $I_{p}\left(t\right)$ at the moment *t* [24,33]

(1) $$\vartheta \left(t\right)=\left(I_{p}\left(0\right)-I_{p}\left(t\right)\right)/I_{p}\left(t\right),$$

where $I_{p}\left(0\right)$ is the intensity of the probe beam at the photodetector plane in the central part of the beam at the time *t* = 0, and $I_{p}\left(t\right)$ is the intensity of the probe beam at the time *t* from the excitation on/off cycle start. The signal is determined by

(2) $$\vartheta \left(t\right)=P_{e}\cdot B\left(t\right)\cdot E_{0}\cdot \varkappa l=P_{e}\cdot B\left(t\right)\cdot E_{0}\cdot 2.303\varepsilon lc,$$

where *P*_e_ is the excitation laser power, ϰ=2.303εc is the linear light absorption coefficient of the sample, *l* is sample path length, ε is molar absorptivity, and *c* is molar concentration of the absorbing substance in the sample. The parameter *B*(*t*) is the time-dependent geometrical constant of the optical schematic [65,66]. The factor $E_{0}$ in Equation (2) is the power-normalized thermooptical constant (full thermooptical constant is $E=P_{e}E_{0}$) characterizing the strength of the thermal lens effect per a unit excitation laser power:

(3) $$E_{0}=\left(-dn/dT\right)/\lambda_{p}k.$$

where *k* is the thermal conductivity, $\lambda_{p}$ is the probe laser wavelength, and the temperature gradient of refractive index $\left(dn/dT\right)\propto \alpha_{T}$ (the volumetric thermal expansion coefficient).

The parameter $t_{c}$ in Equations (2) and (3) is the characteristic time of the thermal lens [24] calculated from the transient curve of thermal lens measurements (Figure 2)

(4) $$t_{c}=\omega_{0e}^{2}/4D_{T}.$$

where, $\omega_{0e}$ is the excitation beam waist radius, *D_T_* is thermal diffusivity.

The steady-state cw thermal lens signal, Equation (1), is

(5) $$\vartheta =\left(I_{p}\left(0\right)-I_{p}\left(\infty \right)\right)/I_{p}\left(\infty \right),$$

and is defined as [33,65,66]

(6) $$\vartheta =BP_{e}E_{0}\varkappa l=BE\cdot 2.303\varepsilon lc=B\theta .$$

where, $\theta \equiv 2.303E_{0}P_{e}\varepsilon lc$ is the thermal lens signal corrected for the steady-state geometry constant *B*(*t* → ∞).

## 5. Conclusions

Thus, using spectrophotometric titration and thermal lens spectrometry, the composition of the ferricytochrome c-cardiolipin complex was determined. Spectrophotometric titration was carried out in the range of micromolar concentrations, where the complex of ferricytochrome *c* and cardiolipin was deposited. Thermal lens measurements were carried out at nanomolar protein concentrations, where the complex is mainly in solution. The ratios of ferricytochrome *c* and cardiolipin by thermal lensing were 50 ± 4 (spectrophotometry, 50 ± 7) at a pH 7.4; 30 ± 4 (33 ± 5) at pH 6.5, and 10 ± 4 (13 ± 3) at pH 3.7. For pH 7.4, a molecular solubility constant of 30 μmol/L was calculated. It was shown that thermal lens spectrometry makes it possible to detect the formation of Cyt *c*–CL complexes directly in solution at a low concentration of ferricytochrome *c* and lipid (10 μM ferricytochrome and below). The results of this study will serve to further clarify the structure and mechanism of functioning of Cyt *c*–CL in the mitochondrial membranes at early stages of apoptosis.

## Figures and Tables

**Table 1:** Molecular solubility, *S*, and TOCL:Cyt *c* molar ratios in the complex at different pH values in an aqueous medium obtained by spectrophotometry; *p* = 0.95, *n* = 3.

Buffer Solution	KH_2_PO_4_, 20 mM				Phthalate, 20 mM
pH	7.4	6.8	5.5	4.1	3.7
TOCL: Cyt *c* molar ratio	50 ± 7	33 ± 5	13 ± 3	14 ± 3	13 ± 4
S, μmol/L	31 ± 4	32 ± 4	11 ± 3	n/a	n/a

**Table 2:** TOCL:Cyt *c* ratios in the complex at different pH values in aqueous solutions (KH_2_PO_4_, 20 mM) by thermal lens spectrometry; *p* = 0.95, *n* = 3.

pH	7.4	6.8	5.5
TOCL:Cyt *c* molar ratio	50 ± 4	30 ± 4	10 ± 4

**Table 3:** Parameters of the thermal lens spectrometer.

Excitation Ar^+^ laser Innova 90-6	Wavelength, λ*_e_* (nm)	514.5
	Power range at the sample (TEM_00_ mode), *P_e_* (mW)	1–200
	Waist radius (µm)	55 ± 3
	Focal distance of the focusing lens (mm)	300
Probe He–Ne laser HRP020	Wavelength, λ*_p_* (nm)	632.8
	Power range at the sample (TEM_00_ mode), *P_p_* (mW)	2
	Waist radius (µm)	25.0
	Focal distance of the focusing lens (mm)	185
Other parameters	Sample optical path *l* (mm)	2.0
	Shutter rate (Hz)	0.5–4
	Geometry constant *B* (Equation (2))	0.378

## Data Availability

The datasets used and/or analyzed during the current study are available from the corresponding author on reasonable request.

## References

[1] Diaz-Quintana A., Perez-Mejias G., Guerra-Castellano A., De la Rosa M.A., Diaz-Moreno I. (2020). Wheel and Deal in the Mitochondrial Inner Membranes: The Tale of Cytochrome c and Cardiolipin. Oxid. Med. Cell. Longev..

[2] Kitt J.P., Bryce D.A., Minteer S.D., Harris J.M. (2017). Raman Spectroscopy Reveals Selective Interactions of Cytochrome c with Cardiolipin That Correlate with Membrane Permeability. J. Am. Chem. Soc..

[3] Vladimirov Y.A., Sarisozen C., Vladimirov G.K., Filipczak N., Polimova A.M., Torchilin V.P. (2017). The Cytotoxic Action of Cytochrome C/Cardiolipin Nanocomplex (Cyt-CL) on Cancer Cells in Culture. Pharm. Res..

[4] Rice M., Wong B., Oja M., Samuels K., Williams A.K., Fong J., Sapse A.M., Maran U., Korobkova E.A. (2021). A role of flavonoids in cytochrome c-cardiolipin interactions. Bioorg. Med. Chem..

[5] Kagan V.E., Tyurin V.A., Jiang J., Tyurina Y.Y., Ritov V.B., Amoscato A.A., Osipov A.N., Belikova N.A., Kapralov A.A., Kini V. (2005). Cytochrome c acts as a cardiolipin oxygenase required for release of proapoptotic factors. Nat. Chem. Biol..

[6] Kagan V.E., Bayir H.A., Belikova N.A., Kapralov O., Tyurina Y.Y., Tyurin V.A., Jiang J., Stoyanovsky D.A., Wipf P., Kochanek P.M. (2009). Cytochrome c/cardiolipin relations in mitochondria: A kiss of death. Free Radic. Biol. Med..

[7] Ripanti F., Di Venere A., Guidi M.C., Romani M., Filabozzi A., Carbonaro M., Piro M.C., Sinibaldi F., Nucara A., Mei G. (2021). The Puzzling Problem of Cardiolipin Membrane-Cytochrome c Interactions: A Combined Infrared and Fluorescence Study. Int. J. Mol. Sci..

[8] Zhan J., Zhang G., Chai X., Zhu Q., Sun P., Jiang B., Zhou X., Zhang X., Liu M. (2021). NMR Reveals the Conformational Changes of Cytochrome C upon Interaction with Cardiolipin. Life.

[9] Vladimirov Y.A., Proskurnina E.V., Izmailov D.Y., Novikov A.A., Brusnichkin A.V., Osipov A.N., Kagan V.E. (2006). Cardiolipin activates cytochrome c peroxidase activity since it facilitates H(2)O(2) access to heme. Biochem. Biokhimiia.

[10] Belikova N.A., Vladimirov Y.A., Osipov A.N., Kapralov A.A., Tyurin V.A., Potapovich M.V., Basova L.V., Peterson J., Kurnikov I.V., Kagan V.E. (2006). Peroxidase activity and structural transitions of cytochrome c bound to cardiolipin-containing membranes. Biochemistry.

[11] Vladimirov Y.A., Demin E.M., Proskurnina E.V., Osipov A.N. (2009). Lipoperoxide radical production during oxidation of cardiolipin in the complex with cytochrome c. Biochem. Suppl. Ser. A Membr. Cell Biol..

[12] Thong A., Tsoukanova V. (2018). Cytochrome-c-assisted escape of cardiolipin from a model mitochondrial membrane. Biochim. Biophys. Acta Biomembr..

[13] Pinto I.F.D., Chaves-Filho A.B., Cunha D.D., Miyamoto S. (2020). Cytochrome c modification and oligomerization induced by cardiolipin hydroperoxides in a membrane mimetic model. Arch Biochem. Biophys..

[14] Vladimirov Y.A., Proskurnina E.V., Alekseev A.V. (2013). Molecular mechanisms of apoptosis. structure of cytochrome c-cardiolipin complex. Biochem. Biokhimiia.

[15] Vladimirov Y.A., Nol’ Y.T., Volkov V.V. (2011). Protein-Lipid Nanoparticles That Determine Whether Cells will Live or Die. Crystallogr. Rep..

[16] Vikulina A.S., Alekseev A.V., Proskurnina E.V., Vladimirov Y.A. (2015). Cytochrome c-Cardiolipin Complex in a Nonpolar Environment. Biochem. Biokhimiia.

[17] Vladimirov G.K., Remenshchikov V.E., Nesterova A.M., Volkov V.V., Vladimirov Y.A. (2019). Comparison of the Size and Properties of the Cytochrome c/Cardiolipin Nanospheres in a Sediment and Non-polar Medium. Biochem. Biokhimiia.

[18] Vladimirov G.K., Vikulina A.S., Volodkin D., Vladimirov Y.A. (2018). Structure of the complex of cytochrome c with cardiolipin in non-polar environment. Chem. Phys. Lipids.

[19] Elmer-Dixon M.M., Bowler B.E. (2017). Site A-Mediated Partial Unfolding of Cytochrome c on Cardiolipin Vesicles Is Species-Dependent and Does Not Require Lys72. Biochemistry.

[20] Kawai C., Prado F.M., Nunes G.L., Di Mascio P., Carmona-Ribeiro A.M., Nantes I.L. (2005). pH-Dependent interaction of cytochrome c with mitochondrial mimetic membranes: The role of an array of positively charged amino acids. J. Biol. Chem..

[21] Elmer-Dixon M.M., Bowler B.E. (2018). Electrostatic Constituents of the Interaction of Cardiolipin with Site A of Cytochrome c. Biochemistry.

[22] Paradisi A., Bellei M., Paltrinieri L., Bortolotti C.A., Di Rocco G., Ranieri A., Borsari M., Sola M., Battistuzzi G. (2020). Binding of S. cerevisiae iso-1 cytochrome c and its surface lysine-to-alanine variants to cardiolipin: Charge effects and the role of the lipid to protein ratio. J. Biol. Inorg. Chem..

[23] Mohammadyani D., Yanamala N., Samhan-Arias A.K., Kapralov A.A., Stepanov G., Nuar N., Planas-Iglesias J., Sanghera N., Kagan V.E., Klein-Seetharaman J. (2018). Structural characterization of cardiolipin-driven activation of cytochrome c into a peroxidase and membrane perturbation. Biochim. Biophys. Acta Biomembr..

[24] Bialkowski S.E., Astrath N.G.C., Proskurnin M.A. (2019). Photothermal Spectroscopy Methods.

[25] Proskurnin M.A., Baudelet M. (2014). Photothermal spectroscopy. Laser Spectroscopy for Sensing.

[26] Brusnichkin A.V., Nedosekin D.A., Galanzha E.I., Vladimirov Y.A., Shevtsova E.F., Proskurnin M.A., Zharov V.P. (2010). Ultrasensitive label-free photothermal imaging, spectral identification, and quantification of cytochrome c in mitochondria, live cells, and solutions. J. Biophoton..

[27] Proskurnin M.A., Khabibullin V.R., Usoltseva L.O., Vyrko E.A., Mikheev I.V., Volkov D.S. (2022). Photothermal and optoacoustic spectroscopy: State of the art and prospects. Physics-Uspekhi.

[28] Santo-Domingo J., Demaurex N. (2012). Perspectives on: SGP symposium on mitochondrial physiology and medicine: The renaissance of mitochondrial pH. J. Gen. Physiol..

[29] Coletta M., Costa H., De Sanctis G., Neri F., Smulevich G., Turner D.L., Santos H. (1997). pH dependence of structural and functional properties of oxidized cytochrome c" from Methylophilus methylotrophus. J. Biol. Chem..

[30] Babul J., Stellwagen E. (1972). Participation of the protein ligands in the folding of cytochrome c. Biochemistry.

[31] Mikheev I.V., Volkov D.S., Proskurnin M.A., Korobov M.V. (2014). Monitoring of Aqueous Fullerene Dispersions by Thermal-Lens Spectrometry. Int. J. Thermophys..

[32] Proskurnin M.A., Usoltseva L.O., Volkov D.S., Nedosekin D.A., Korobov M.V., Zharov V.P. (2021). Photothermal and Heat-Transfer Properties of Aqueous Detonation Nanodiamonds by Photothermal Microscopy and Transient Spectroscopy. J. Phys. Chem. C.

[33] Khabibullin V.R., Usoltseva L.O., Galkina P.A., Galimova V.R., Volkov D.S., Mikheev I.V., Proskurnin M.A. (2023). Photothermal Properties and Measurement Precision of Nanostructures in Aqueous Solutions by Transient and Steady-State Thermal-Lens Spectrometry. Physchem.

[34] Jain R., Kumar R., Kumar S., Chhabra R., Agarwal M.C., Kumar R. (2015). Analysis of the pH-dependent stability and millisecond folding kinetics of horse cytochrome c. Arch. Biochem. Biophys..

[35] Wilkinson J.A., Silvera S., LeBlanc P.J. (2021). The effect of cardiolipin side chain composition on cytochrome c protein conformation and peroxidase activity. Physiol. Rep..

[36] Milorey B., Schweitzer-Stenner R., Kurbaj R., Malyshka D. (2019). pH-Induced Switch between Different Modes of Cytochrome c Binding to Cardiolipin-Containing Liposomes. ACS Omega.

[37] Pandiscia L.A., Schweitzer-Stenner R. (2014). Salt as a catalyst in the mitochondria: Returning cytochrome c to its native state after it misfolds on the surface of cardiolipin containing membranes. Chem. Commun..

[38] Oellerich S., Lecomte S., Paternostre M., Heimburg T., Hildebrandt P. (2004). Peripheral and Integral Binding of Cytochrome c to Phospholipids Vesicles. J. Phys. Chem. B..

[39] Kuroda Y., Kidokoro S., Wada A. (1992). Thermodynamic characterization of cytochrome c at low pH. Observation of the molten globule state and of the cold denaturation process. J. Mol. Biol..

[40] Battistuzzi G., Bortolotti C.A., Bellei M., Di Rocco G., Salewski J., Hildebrandt P., Sola M. (2012). Role of Met80 and Tyr67 in the low-pH conformational equilibria of cytochrome c. Biochemistry.

[41] Haines H. (2009). A new look at Cardiolipin. Biochim. Biophys. Acta.

[42] Olofsson G., Sparr E. (2013). Ionization constants pKa of cardiolipin. PLoS ONE.

[43] Parui P.P., Sarakar Y., Majumder R., Das S., Yang H., Yasuhara K., Hirota S. (2019). Determination of proton concentration at cardiolipin-containing membrane interfaces and its relation with the peroxidase activity of cytochrome c. Chem. Sci..

[44] Brown L.R., Wuthrich K. (1977). NMR and ESR studies of the interactions of cytochrome c with mixed cardiolipin-phosphatidylcholine vesicles. Biochim. Biophys. Acta.

[45] Pinheiro T.J., Cheng H., Seeholzer S.H., Roder H. (2000). Direct evidence for the cooperative unfolding of cytochrome c in lipid membranes from H-(2)H exchange kinetics. J. Mol. Biol..

[46] Muenzner J., Pletneva E. (2013). Structural Transformations of Cytochrome c upon Interaction with Cardiolipin. Chem. Phys. Lipids.

[47] Hong Y., Muenzner J., Grimm S.K., Pletneva E.V. (2012). Origin of the conformational heterogeneity of cardiolipin-bound cytochrome C. J. Am. Chem. Soc..

[48] Gorbenko G.P., Molotkovsky J.G., Kinnunen P.K. (2006). Cytochrome C interaction with cardiolipin/phosphatidylcholine model membranes: Effect of cardiolipin protonation. Biophys. J..

[49] Domanov Y.A., Molotkovsky J.G., Gorbenko G.P. (2005). Coverage-dependent changes of cytochrome c transverse location in phospholipid membranes revealed by FRET. Biochim. Biophys. Acta.

[50] Beales P.A., Bergstrom C.L., Geerts N., Groves J.T., Vanderlick T.K. (2011). Single vesicle observations of the cardiolipin-cytochrome C interaction: Induction of membrane morphology changes. Langmuir.

[51] Li M., Mandal A., Tyurin V.A., DeLucia M., Ahn J., Kagan V.E., van der Wel P.C.A. (2019). Surface-Binding to Cardiolipin Nanodomains Triggers Cytochrome c Pro-apoptotic Peroxidase Activity via Localized Dynamics. Structure.

[52] Gohil V.M., Greenberg M.L. (2009). Mitochondrial membrane biogenesis: Phospholipids and proteins go hand in hand. J. Cell. Biol..

[53] Zinser E., Sperka-Gottlieb C.D., Fasch E.V., Kohlwein S.D., Paltauf F., Daum G. (1991). Phospholipid synthesis and lipid composition of subcellular membranes in the unicellular eukaryote Saccharomyces cerevisiae. J. Bacteriol..

[54] Fukuzawa K., Iemura M., Tokumura A. (1996). Lipid peroxidation in egg phosphatidylcholine liposomes: Comparative studies on the induction systems Fe^2+^/ascorbate and Fe^(3+)^-chelates/xanthine-xanthine oxidase. Biol. Pharm. Bull..

[55] Chasovnikova L.V., Formaziuk V.E., Sergienko V.I., Vladimirov Iu A. (1991). Peroxidation of phosphatidylcholine and cholesterol in mixed monolayers. Biofizika.

[56] van Duijn G., Verkleij A.J., de Kruijff B. (1984). Influence of phospholipid peroxidation on the phase behavior of phosphatidylcholine and phosphatidylethanolamine in aqueous dispersions. Biochemistry.

[57] Kubo K., Sekine S., Saito M. (2003). Docosahexaenoic acid-containing phosphatidylethanolamine in the external layer of liposomes protects docosahexaenoic acid from 2,2'-azobis(2-aminopropane)dihydrochloride-mediated lipid peroxidation. Arch. Biochem. Biophys..

[58] Butler E.S.O., Mazerik J.N., Cruff J.P., Sherwani S.I., Weis B.K., Marsh C.B., Raghavamenon A.C., Uppu R.M., Schmid H.H., Parinandi N.L. (2010). Lipoxygenase-catalyzed phospholipid peroxidation: Preparation, purification, and characterization of phosphatidylinositol peroxides. Meth. Mol. Biol..

[59] Jiang J., Serinkan B.F., Tyurina Y.Y., Borisenko G.G., Mi Z., Robbins P.D., Schroit A.J., Kagan V.E. (2003). Peroxidation and externalization of phosphatidylserine associated with release of cytochrome c from mitochondria. Free Radic. Biol. Med..

[60] Viani P., Cervato G., Fiorilli A., Rigamonti E., Cestaro B. (1990). Studies on peroxidation processes of model membranes and synaptosomes: Role of phosphatidic acid. Chem. Phys. Lipids.

[61] Tyurina Y.Y., Shvedova A.A., Kawai K., Tyurin V.A., Kommineni C., Quinn P.J., Schor N.F., Fabisiak J.P., Kagan V.E. (2000). Phospholipid signaling in apoptosis: Peroxidation and externalization of phosphatidylserine. Toxicology.

[62] Matsura T., Serinkan B.F., Jiang J., Kagan V.E. (2002). Phosphatidylserine peroxidation/externalization during staurosporine-induced apoptosis in HL-60 cells. FEBS Lett..

[63] Jiang J., Kini V., Belikova N., Serinkan B.F., Borisenko G.G., Tyurina Y.Y., Tyurin V.A., Kagan V.E. (2004). Cytochrome c release is required for phosphatidylserine peroxidation during Fas-triggered apoptosis in lung epithelial A549 cells. Lipids.

[64] Khabibullin V.R., Franko M., Proskurnin M.A. (2023). Accuracy of Measurements of Thermophysical Parameters by Dual-Beam Thermal-Lens Spectrometry. Nanomaterials.

[65] Shen J., Lowe R.D., Snook R.D. (1992). A model for cw laser-induced mode-mismatched dual-beam thermal lens spectrometry. Chem. Phys..

[66] Baesso M.L., Shen J., Snook R.D. (1992). Time-resolved thermal lens measurement of thermal diffusivity of soda—Lime glass. Chem. Phys. Lett..

